# Intravenous immunoglobulins in infantile dyshidrosiform bullous pemphigoid refractory to steroids and dapsone

**DOI:** 10.1111/dth.15826

**Published:** 2022-09-27

**Authors:** Gianmaria Viglizzo, Astrid Herzum, Emanuele Cozzani, Corrado Occella, Aurora Parodi

**Affiliations:** ^1^ Section of Dermatology IRCCS Istituto Giannina Gaslini Genoa Italy; ^2^ Section of Dermatology, Department of Health Sciences (DISSAL) University of Genoa, IRCCS San Martino Polyclinic Hospital Genoa Italy

Dear Editor,

Dyshidrosiform bullous pemphigoid (DBP) is a rare variant of bullous pemphigoid (BP), autoimmune blistering disease mostly affecting the elderly.

DBP was described by Levine et al. in 1979, as pruritic blisters on hands and feet, resembling pompholyx, often generalizing to other body sites.^1^, ^2^

In pediatric population, dyshidrosiform presentation of BP is a constant initial BP manifestation in infants, while in adults the incidence of DBP ranges from 3.5% to 28%.^2^, ^3^

Steroids represent the primary treatment of BP, adding steroid‐sparing agents when needed. Almost all adults and 93% of infants achieve remission.^2^, ^3^ Recurrences are reported in 12% of infants, especially after rapid steroid tapering.

We report a case of DBP, successfully treated with intravenous immunoglobulins (IVIGs) after ineffective classical steroid therapy.

A 1‐month‐old boy who developed pruritic blisters on his hands and feet, progressively generalizing (Figure 1), was referred to the dermatology clinic after ineffective impetigo treatment with amoxiclavulanate. At clinical examination, mucosae were spared, Nikolsky sign was negative, and generalized pruritic, tense, hemorrhagic bullae were evidenced raising suspicion of BP. Laboratory findings revealed leukocytosis (58,000/mm^3^), eosinophilia (24,000/mm^3^), and thrombocytosis (1,000,000/mm^3^). Skin biopsy was performed for histopathological examination and direct immunofluorescence (DIF), revealing respectively multiple eosinophils in the superficial dermis and in subepidermal clefts and linear staining of IgG and C3 along the dermoepidermal junction. Enzyme‐linked immunosorbent assay (ELISA, commercial‐kits EuroimmunAG) evidenced IgG anti‐BP180‐antigen (234.5 U/ml) and IgG anti‐BP230‐antigen (22.8 U/ml) (normal values <20 U/ml).

**FIGURE 1:**
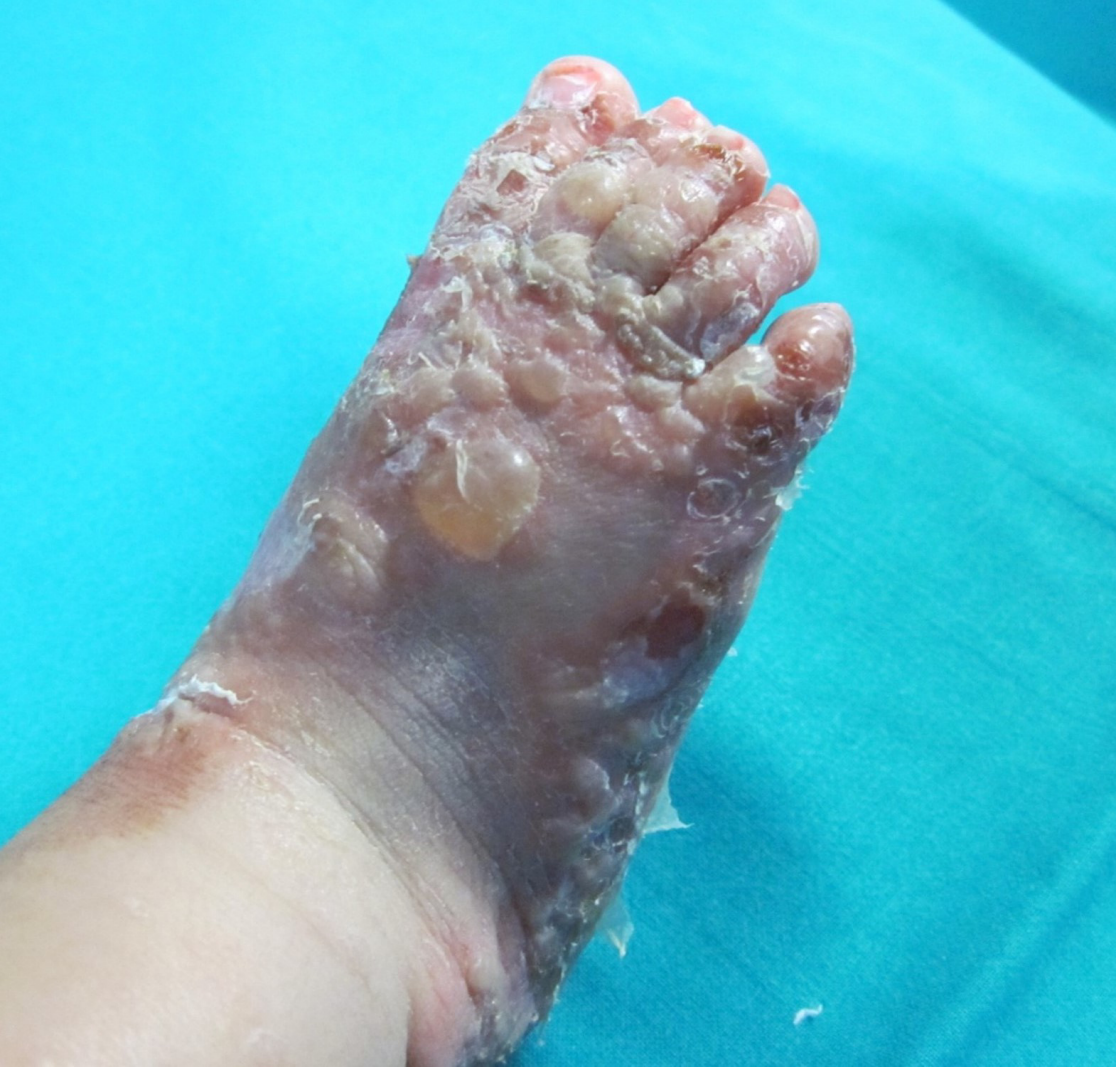
Clinical presentation of dyshidrosiform bullous pemphigoid in a 1‐month‐old boy: tense hemorrhagic bullae, partially ruptured and forming erosions and crusts, on the patient's foot

Infantile BP (IBP) was diagnosed. Oral betamethasone was administered (0.2 mg/kg/day), obtaining clinical remission within 1 month; it was progressively tapered. A relapse after tapering was controlled with prednisone (25 mg/day) and dapsone (50 mg/day), but weight gain and insomnia forced to taper steroids, worsening BP. IVIGs were added (1 g/kg/day, 2‐day‐course, monthly, 6 months) with immediate clinical improvement, allowing tapering of prednisone (5 mg/day) and dapsone (25 mg/day) within 4 months, as remission was obtained after the fourth IVIGs administration. Dapsone (25 mg/day) was still administered for 1 year. During 2‐year‐follow‐up no further relapses were reported; ELISA IgG autoantibodies were negative.

BP represents the most common bullous autoimmune disease in adults, whereas pediatric BP is rarer.^4^ Acral dyshidrotic BP is the major presentation in infants (100% of IBP), with progressive generalization in 91%. Noteworthy, most DBP patients present tense bullae on hands and feet, resembling dyshidrosiform dermatitis, often evolving into haemorrhagic bullae “haemorrhagic pompholyx,” mostly generalizing, as in our case.

Though no official treatment guidelines exist for IBP, steroids are administered as first‐line therapy (97% of cases), adding steroid‐sparing agents in 60% of patients. Overall, 93% obtain remission.^3^, ^5^ Dapsone is the mostly used steroid sparing agent (70%), effectively inhibiting neutrophilic chemotaxis and activity, and being well tolerated.^3^, ^6^

Occasionally, IBP may be refractory to systemic corticosteroids and dapsone, as in the present case: IVIGs (2 g/kg/4w) may be of aid in these circumstances, rapidly and safely leading to clinical improvement.^2^, ^3^

In literature cases of IBP treated with IVIGs are anecdotal, reporting variable dosages, (200–1000 mg/kg/day), number (1–7), duration (1–5 days) of infusion‐courses and intervals between courses (2–5 weeks). Mostly, administered doses corresponded to the IVIG regimen of adults (400 mg/kg/day, 5 days) for autoimmune bullous diseases comprising BP.^7^ Reported cases mostly reached clinical remission after IVIG administration, permitting rapid taper of corticosteroids. No severe adverse effects attributable to IVIGs were reported.^7^

Conclusively, IVIG are a well‐tolerated, effective, broad‐spectrum therapeutic alternative for severe, therapy‐refractory immune diseases as BP.^5^ However, the high costs limit IVIGs' use to selective indications.^8^

Still, in difficult‐to‐treat, fragile BP patients, as the presented one‐month‐old infant, IVIGs' safety and therapeutic potency highly favor their administration, which is mostly resolutive.^5^, ^7^

## AUTHOR CONTRIBUTIONS

Gianmaria Viglizzo, Astrid Herzum, Emanuele Cozzani, Corrado Occella, and Aurora Parodi contributed equally to the manuscript and read and approved the final version of the manuscript.

## CONFLICT OF INTEREST

The authors certify that there is no conflict of interest with any financial organization regarding the material discussed in the manuscript.

## ETHICS STATEMENT

The present research study complies with the guidelines for human studies and includes evidence that the research study was conducted ethically in accordance with the World Medical Association Declaration of Helsinki. Written informed consent to publish the case (including publication of images) was obtained.

## Data Availability

The data that support the findings of this study are available from the corresponding author upon reasonable request.
